# Changes in Location

**Published:** 1916-07

**Authors:** 


### Changes in Location.

Dr. J. T. Pue and family, of Sweetwater, have moved to Merkel, where they will make their future home.

Dr. Thatcher Tinkle, of Timpson, has moved to Cameron, where he will assist Dr. J. B. Turner in the practice of medicine.

Dr. A. J. Caldwell has moved from San Antonio to Amarillo.

Dr. and Mrs. O. H. Radkey have moved to Austin to make their future home. They have just completed a beautiful new home on Speedway.

